# Expression of Tight Junction Proteins Is Altered in Bladder Cancer

**DOI:** 10.1155/2020/6341256

**Published:** 2020-11-16

**Authors:** Bangbin Chen, Renge Bu, Xuewen Xu

**Affiliations:** Department of Urology, Shengjing Hospital of China Medical University, Shenyang, Liaoning, China 110004

## Abstract

Bladder cancer (BC) is one of the tumors which occur most frequently in urological system, but less is known about the expression of tight junction proteins and its clinical significance in BC. In this study, expression of claudin-4, zonula occludens-1 (ZO-1) and zonula occludens-1 nucleic acid-binding protein (ZONAB), in BC tissues, adjacent nontumor tissue (ANTT), and BC cell lines was examined by Western blotting, semiquantitative RT-PCR, and immunohistochemistry, and then, the clinical significance of these proteins was investigated. The mRNA and protein expression of ZONAB were significantly upregulated, while those of ZO-1 was significantly downregulated in some BC cell lines and tissues in comparison with nontumor urothelial cell lines and ANTT. High expression rate of ZO-1 and ZONAB had negative correlation in BC tissues and was also correlated with muscle-invasive lesions in BC tissues. In conclusion, the expression of tight junction proteins is significantly altered in BC and ZO-1, and ZONAB interaction might be involved in BC development.

## 1. Introduction

Bladder cancer (BC) is one of the tumors which occur most frequently in the urological system. Although there are more and more treatments for BC, the recurrence rate and mortality rate are still relatively high, which makes it more urgent to explore the mechanism of tumorigenesis [1]. Intercellular adhesion is the first barrier against development of solid tumors [2]. As an important intercellular junction, tight junction turns out to be important in inhibition of neoplastic proliferation and metastasis [3, 4]. Tight junction is a protein strand that seals the gap between adjacent cells and separates the lateral intercellular space from apical lumen of the epithelium [5]. It also participates in intercellular signaling and involves in several biological processes [6–8]. Tight junctions consist of three groups of proteins: integral membrane proteins, plaque anchoring proteins, and tight junction regulatory proteins [9, 10]. The extracellular domain of the membrane protein binds to corresponding tight junction protein of adjacent cells [11, 12]. The intracellular domain binds to the plaque anchoring protein which is located on the cell membrane's inner surface and binds to the cytoskeletal protein [13]. The integrity of tight junctional component is vital for the maintenance of cell polarity, morphology, and barrier function [10, 14, 15].

Altered expression of tight junction proteins attenuates the barrier function against cancer development [2, 16, 17]. In order to define the antitumor function of tight junction, it is necessary to detect the expression level of those proteins in malignant tumors [18]. Claudin-4 is one important integral membrane protein belonging to tight junction. Claudin-4 expression is altered in several solid tumors. For example, its expression in triple-negative breast cancer is markedly upregulated and downregulated in colorectal cancer [19, 20]. Overexpression of that in ovarian cancer predicts a poor 5-year survival rate [21]. ZO-1 is one of plaque anchoring proteins of tight junction and its abnormal expression is also observed in some malignancies. ZO-1 expression is significantly decreased in non-small cell lung cancer [22]. It is also involved in ZIP4 initiated pancreatic cancer invasion [23]. ZONAB as a tight junction regulatory protein is involved in estrogen-induced breast cancer metastasis [24]. ZONAB is also involved in the development of ethanol-induced gastric cancer [25]. Claudin-4, ZO-1, and ZONAB serve as important proteins of tight junction, and their normal expression is vital for the barrier function, particularly the process of cancer cell proliferation as well as metastasis. However, the expression profile of these proteins in BC has not been systematically examined.

Based on the findings of claudin-4, ZO-1, and ZONAB expression in other solid tumors, we speculate that their expression in BC is also altered. We expect to have further understanding on the integrity of tight junction structure and its correlation with clinical manifestation in BC. By doing so, we provide a basis for defining the specific character of tight junction component in the development of BC. In order to verify our hypothesis, we examined BC tissues and ANTT of their claudin-4, ZO-1, and ZONAB expression and analyzed their correlation with each other as well as clinicopathological factors.

## 2. Materials and Methods

### 2.1. Tissue Samples

53 paired BC and ANTT specimens were obtained from patients undergoing surgery between January 2016 and January 2017 at the Department of Urology, Shengjing Hospital Affiliated to China Medical University, Shenyang, China. All patients were diagnosed by pathology with bladder urothelial carcinoma. BC tissues and ANTT (tumor-free bladder tissues taken 2 cm away from the edge of the tumor) specimens were obtained and washed with RNase-free 0.9% sodium chloride to eliminate blood intraoperatively within 5 minutes after resection. Nontumor tissues were removed from tumor specimens, and smooth muscle from ANTT. Partial samples for RNA extraction were preserved, stored in liquid nitrogen at once, and then transferred to a -80°C deep-freeze. Histological examination confirmed that more than 80% of the frozen BC tissue consisted of tumor cells for RNA extraction. The remaining sample was fixed in 10% formalin for pathological examination and immunohistochemical staining.

### 2.2. Cell Lines

The human noncancer urothelial cell line sv-huc-1 and bladder urothelial carcinoma cell lines 5637, UM-UC-3, and T24 were obtained through the Cell Bank of Type Culture Collection of the Chinese Academy of Sciences (Shanghai). We got sv-huc-1 cells cultured in F12K, UM-UC-3 cells in DMEM, T24, and 5637 cells in RPMI 1640 medium all with 10% heat-inactivated fetal bovine serum (FBS) at 37°C under 5% CO2.

### 2.3. Western Blotting

RIPA cell lysate in suitable volume was added to the cells. After centrifugation, by the bicinchoninic acid (BCA) protein concentration assay kit (Beyotime, Shanghai, China), we got the concentration of the protein in the supernate. Before being diluted by a 5× Loading Buffer, the samples were boiled with water bath lasting for 5 minutes. Then, we used SDS-PAGE to separate the protein and transferred them to a PVDF membrane (Millipore, USA). Since being incubated with primary antibody, including mouse anti-*β*-actin monoclonal antibody (diluted 1 : 1000; Cell Signaling Technology), mouse anti-claudin-4 monoclonal antibody (diluted 1 : 500; Invitrogen), rabbit anti-ZO-1 polyclonal antibody (diluted 1 : 250; Invitrogen), rabbit anti-ZONAB polyclonal antibody (diluted 1 : 1000; Invitrogen), and goat secondary antibody conjugated to horseradish peroxidase (diluted 1 : 5000; Sangon, Shanghai, China), ECL luminescence solution (Sigma, St. Louis, MO) was added as luminous substrate, and the band intensity was analyzed by a digital scanning imaging system.

### 2.4. RNA Expression Analysis

For the detection of RNA expression, we used the method of semiquantitative RT-qPCR. We washed the collected cell samples twice through PBS, froze tissue samples in liquid nitrogen, and then ground them to powder. By adding TRIzol reagent, we could extract the total RNA. The extracted RNA absorbance was measured using a spectrophotometer to calculate concentration. By reverse transcription through the PrimeScript RT Master Mix kit (Takara Biotechnology Co., Ltd., Japan), total RNA was used to develop cDNA. Real-time PCR was conducted with cDNA as the template using the SYBR Premix Ex Taq II kit (Takara Biotechnology Co., Ltd., Japan) within the AriaMx real-time fluorescence PCR system (Agilent, USA). PCR amplification conditions were as follows: 95°C denaturation 30 s, 45 PCR cycles (95°C 5 s; 60°C 20 s), 65°C extended 10 s, and 65°C fluorescence signal detection, to obtain the standard curve. We took GAPDH mRNA as internal control and water as a negative control. Primer sequences turned out to be as follows: claudin-4 upstream: 5′-AGCTCTGTGGCCTCAGGACTCT-3′, downstream: 5′-CAGTGATGAATAGCTCTTCTTAAATTACAA-3′; ZO-1 upstream: 5′-GGATGTTTATCGTCGCATTGTA-3′, downstream: 5′-AAGAGCCCAGTTTTCCATTGTA-3′; ZONAB upstream: 5′-GCTGGGGAGGAGGAGGA-3′, downstream: 5′-CTGTTGGGATGGGGTAAGAC-3′; and GAPDH upstream: 5′-TCCCTGAGCTGAACGGGAAG-3′, downstream: 5′-GGAGGAGTGGGTGTCGCTGT-3′. The relative expression level of the each gene was obtained according to the standard curve. Statistical analysis was performed using the 2^-*ΔΔ*Cq^ method.

### 2.5. Immunohistochemistry

Before putting BC tissues and ANTT in sodium citrate in microwave for antigen retrieval, we fixed them in formalin, embedded them in paraffin, sectioned them at 5 *μ*m thick, and then dewaxed and rehydrated them. The slides were added by a nonspecific stain blocking agent, and then incubated them at room temperature for 30 minutes. Anti-human claudin-4 primary antibody (1 : 100; Zymed, San Francisco, CA, USA) or anti-human ZO-1 primary antibody (1 : 300, Abcam, Cambridge, MA) or anti-human ZONAB primary antibody (1: 200, Thermo Fisher, Shanghai, China) was added and incubated for a whole night at 4°C. We took PBS for slide washing, and then incubated them with secondary antibody (1 : 100, Gene Tech, Shanghai, China) for 30 minutes. After washing off excessive secondary antibody, we incubated them with Streptavidin-ABC, got them developed by diaminobenzaminidine (DAB), washed, counterstained, dehydrated, dried, and finally mounted by neutral balsam. Under the microscope, we observed and photographed tissue morphology and compared them with sections which were already known positive. Two pathologists were invited to evaluate the results blindly. For each section, 5 high power fields (original magnification ×400) were selected at random, and in every field, 200 cells were observed. We acquire semiquantitative results by confirming staining intensity and scale. Positive cells were defined as follows: any cell whose nucleus or cytoplasm were stained dark brown or yellow. Cell staining intensity score: 0 for no staining, 1 for weak staining, 2 for moderate staining, and 3 for strong staining. The cell staining scale score was as follows: 0 for positive cells ≤5%, 1 for 6-25%, 2 for 6-50%, 3 for 51-75%, 4 for >75%. The composite staining score (cell staining intensity score + cell staining scale score) is as follows: 2 points were considered negative staining (low staining), 3 to 4 points were considered moderate staining, and 5 to 6 points were considered high staining.

### 2.6. Statistical Analysis

Data analysis was conducted using a statistical program by the GraphPad Prism 7 software, plotting box, and whisker graphs as well as stacked bars. We conducted three independent and repeated trials, acquired results of each assay, then had them expressed as whiskers (the minimal and the maximal value) as well as the boxes (the interquartile range of the measure). For comparing those numerical data from the two groups, we performed Student's *t*-test. One-way ANOVA together with post hoc Tukey's multiple comparison test was conducted as well for comparing the statistical difference between more than two groups. As for categorical data, Fisher's exact test could show their difference. Correlation analysis of the data was processed using Spearman's rank order correlation coefficient.

## 3. Results

The tight junction protein expression is often changed in solid tumors [19–25]. To identify their expression pattern in BC, we detected claudin-4, ZO-1, and ZONAB protein level in 1 human noncancer urothelial cell line sv-huc-1 with 3 human BC cell lines 5637, UM-UC-3, and T24 through Western blotting. The findings showed the tight junction protein expression within BC cells is altered. The mRNA expression of tight junction proteins is also altered both in BC cell lines and tissues. Claudin-4, ZO-1, and ZONAB mRNA expression was detected in sv-huc-1, 5637, UM-UC-3, and T24 cell lines through real-time PCR to verify those findings from Western blotting. The results of RNA expression analysis were consistent with the observations in the protein expression analysis.

To validate the result in these cell lines, we detected claudin-4, ZO-1, and ZONAB mRNA level in 53 paired ANTT and BC tissue samples. Data were gathered form 42 males and 11 females with a mean age of 67.3 (44 to 89). Thirty cases had low-grade urothelial carcinoma while the rest, 23, were in high-grade due to tumor grading. Tumor staging was carried out following TNM staging and the WHO classification system [26, 27]. There were 30 cases in stage Ta + T1 and 23 cases in stage T2 + T3. Nine of them were with LN metastasis, and 3 of them were with distant metastasis. None of the patients had been treated with any radiotherapy, chemotherapy, or vesicle instillation before surgery. The average diameter of resected tumors was2.5 ± 1.4cm. The high expression rate of claudin-4 and ZONAB is elevated, while that of ZO-1 is decreased in BC samples.

In order to identify the clinical significance of altered tight junction protein expression, the correlation between claudin-4, ZO-1, ZONAB expression, and clinicopathological factors was analyzed.

**Figure 1:**
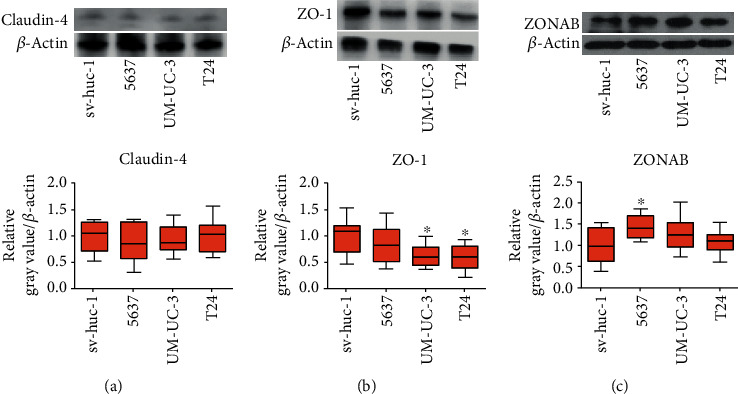
ZO-1 expression is downregulated and ZONAB expression is upregulated in some bladder cancer cell lines. The expression of claudin-4 (a), ZO-1 (b), and ZONAB (c) protein in noncancer cell line (sv-huc-1) and bladder cancer cell lines (5637, UM-UC-3, and T24) was detected by Western blot. The relative grey value adjusted to the internal control *β*-actin and then standardized to the value of sv-huc-1 is shown by box and whisker plots. The whiskers represent the minimal or the maximal gray value, and the boxes span the interquartile range of measurements for cell lines with the mean value of 10 replicates (*n* = 10). ∗*P* < 0.05, one-way ANOVA, post hoc Tukey's test.

**Figure 2:**
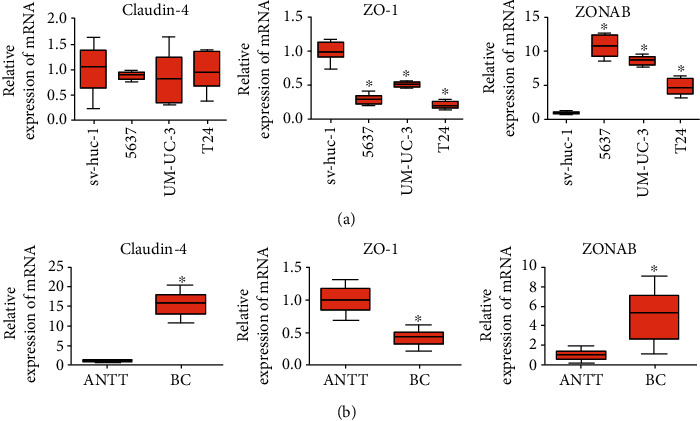
The expression of ZO-1 mRNA is downregulated and ZONAB mRNA is upregulated in bladder cancer (BC) cell lines and BC tissues. (a) The expression of claudin-4, ZO-1, and ZONAB mRNA in noncancer cell line (sv-huc-1) and bladder cancer cell lines (5637, UM-UC-3, and T24) was detected by real-time PCR. Relative expression adjusted to the reference gene GAPDH and then standardized to sv-huc-1 is shown by box and whisker plots. The whiskers represent the minimal or the maximal value and the boxes spans the interquartile range of measurements for cell lines with the mean value of 10 replicates (*n* = 10). ∗*P* < 0.05, one-way ANOVA, post hoc Tukey's test. (b) The expression of claudin-4, ZO-1, and ZONAB mRNA in BC tissue and adjacent nontumor tissues (ANTT) was detected by real-time PCR. Relative expression adjusted to the reference gene GAPDH and then standardized to the value of ANTT is shown by box and whisker plots. The whiskers represent the minimal or the maximal value, and the boxes span the interquartile range of measurements for 53 patients with the mean value of 3 replicates (*n* = 53).∗*P* < 0.05, Student's *t*-test.

**Figure 3:**
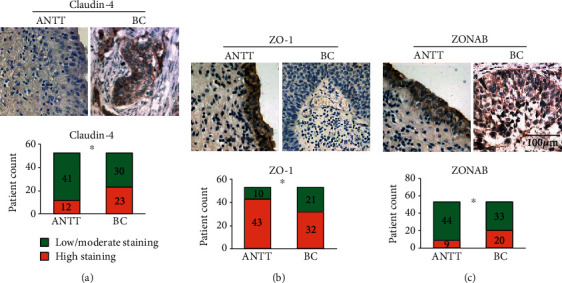
The expression of tight junction proteins is altered in bladder cancer (BC) tissues. Claudin-4 (a), ZO-1 (b), and ZONAB (c) expression was detected by immunohistochemical staining in adjacent nontumor tissues (ANTT) and BC tissues (original magnification ×400. Scale bar, 100 *μ*m). The patient counts on high staining and low/moderate staining of tight junction protein in ANTT and BC is shown by stack bar plots (*n* = 53). ∗*P* < 0.05, Fisher's exact test.

As regards claudin-4, no significant difference was found between BC cell lines (5637: 0.88 ± 0.38, UM-UC-3: 0.93 ± 0.27, and T24: 0.99 ± 0.31) and that of noncancer urothelial cell line sv-huc-1 (1.0 ± 0.29, *P* > 0.05, Figure 1(a)). The claudin-4 mRNA expression showed no significant change in BC cell lines (5637: 0.88 ± 0.08, UM-UC-3: 0.84 ± 0.46, and T24: 0.98 ± 0.36) over that of noncancer urothelial cell line sv-huc-1 (1.0 ± 0.47, *P* > 0.05, Figure 2(a)) but was significantly upregulated in BC tissue (15.50 ± 2.76) over that of ANTT (1.0 ± 0.19, *P* < 0.05, Figure 2(b)). Immunohistochemical staining of the claudin-4 protein showed strong cytoplasmic staining in ANTT and BC tissues (Figure 3(a)). The rate of high expression levels of claudin-4 was 22.6% in ANTT (12/53) and 43.4% in BC tissues (23/53, *P* < 0.05, Figure 3(a)).

ZO-1 protein expression was markedly decreased in BC cell lines (UM-UC-3: 0.63 ± 0.22, T24: 0.58 ± 0.24) over that of noncancer urothelial cell line sv-huc-1 (1.0 ± 0.33, *P* < 0.05, Figure 1(b)). ZO-1 mRNA expression appeared to significantly decrease in BC cell lines (5637: 0.29 ± 0.07, UM-UC-3: 0.51 ± 0.04, and T24: 0.21 ± 0.05) over that of noncancer urothelial cell line sv-huc-1 (1.0 ± 0.13, *P* < 0.05, Figure 2(a)) and to significantly decrease in BC tissue (0.42 ± 0.12) over that of ANTT (1.0 ± 0.19, *P* < 0.05, Figure 2(b)). There was strong ZO-1 membranous staining in ANTT and weak cytoplasmic staining in BC tissues (Figure 3(b)). The rate of high expression levels of ZO-1 was 81.1% in ANTT (43/53) and 60.4% in BC tissues (32/53, *P* < 0.05, Figure 3(b)). Immunohistochemical staining showed that a high expression rate of ZO-1 existed in 10/23 (43.5%) muscle-invasive BC tissues, and a negative correlation between high expression rate of ZO-1 and muscle-invasiveness was found in BC tissues (Spearman's rank order correlation coefficient: -0.303, *P* < 0.05, Table 1).

The ZONAB protein expression was in significant upregulation in BC cell line (5637: 1.44 ± 0.29) compared to noncancer urothelial cell line sv-huc-1 (1.0 ± 0.41, *P* < 0.05, Figure 1(c)). Its mRNA expression of BC cell lines appeared to significantly upregulate (5637: 10.90 ± 1.53, UM-UC-3: 8.66 ± 0.65, T24: and 4.82 ± 1.23) compared to noncancer urothelial cell line sv-huc-1 (1.0 ± 0.20, *P* < 0.05, Figure 2(a)) and to significantly increase in BC tissue (4.97 ± 2.48) over that of ANTT (1.0 ± 0.48, *P* < 0.05, Figure 2(b)). Strong membranous staining of the ZONAB protein in ANTT and strong cytoplasmic staining in BC tissues were observed (Figure 3(c)). The rate of high expression levels of ZONAB was 17.0% in ANTT (9/53) and 37.7% in BC tissues (20/53, *P* < 0.05, Figure 3(c)). ZONAB is negatively correlated to ZO-1 as regards to the high expression rate in BC tissues, and they are correlated to muscle-invasive BC lesions. The negative correlation between the expression of ZO-1 and ZONAB suggests that upregulation of ZONAB is correlated with the attenuation of tight junction function in BC, as ZO-1 is a critical anchoring protein for intact tight junction function. Furthermore, immunohistochemical staining of claudin-4, ZO-1, and ZONAB showed that a positive correlation between high expression rate of ZONAB and muscle-invasiveness was observed in BC tissues (Spearman's rank order correlation coefficient: 0.418, *P* < 0.05, Table 1).

These findings suggest that the tight junction component is involved in the tumorigenesis of BC. No significant correlation between tight junction proteins and other pathological factors like gender, age, local LN, and distant metastasis was found (*P* > 0.05, Table 1).

## 4. Discussion

Extensive expression alteration of tight junction proteins and its tumorigenic character were observed within solid tumors [2, 28]. We observed the change of tight junction protein expression in BC.

High expression rate of ZONAB is negatively correlated with ZO-1, and high expression rate of ZO-1 and ZONAB is correlated with muscle-invasiveness of BC.

Claudin-4 belongs to the claudin family and is encoded by CLDN4 gene. Knockdown of claudin-4 can inhibit cell motility [29]. CLDN4 gene is removed in the Williams-Beuren syndrome, and its abnormal expression is observed in several malignancies [30]. The expression of claudin-4 is distinctly reduced in nonmelanoma skin cancer and colorectal cancer [20, 31], while its expression is upregulated in several malignancies which might be used as diagnostic and therapeutic approach. Michl et al. found that cluadin-4 may be a potential therapy for cancer by acting as a specific receptor for *Clostridium perfringens* enterotoxin (CPE) [32]. Neesse et al. developed a novel C-CPE-Cy5.5-mediated imaging method for pancreatic tumors [33]. We observed the upregulation of claudin-4 in BC tissues. Claudin-4 expression has prognostic value for some tumors. For example, overexpression of claudin-4 suggested shorter 5-year progression-free survival and overall survival of ovarian cancer [34], while low expression of claudin-4 indicated a shorter disease-free survival of breast carcinoma in situ [35].

ZO-1 is encoded by TJP1 gene and serves in regulation of adherent. Altered expression of ZO-1 is found in many solid tumors. Its expression is correlated with malignant phenotypes of gastrointestinal stromal tumor [36]. ZO-1 redistribution also regulates cell dissociation in pancreatic cancer [37]. We observed that ZO-1 was downregulated both in some BC cell lines and tissues, and high ZO-1 expression was negatively correlated with muscle invasion of BC, which means that downregulation of ZO-1 might be involved in BC invasion. Regular ZO-1 expression is critical for maintaining barrier function against tumor development. Disrupted ZO-1 expression promotes dissociation of pancreatic cancer cells [37]. High expression of ZO-1 is related to better prognosis of non-small cell lung cancer [22], while decreased expression of ZO-1 in colorectal cancer connects with liver metastasis closely [38].

ZONAB is encoded by the YBX3 gene, and it has double-stranded DNA-binding activity and transcription factor activity via binding to the GM-CSF promoter, as well as activity binding to intact mRNA or RNA fragments containing the 5′-UCCAUCA-3′ sequence [39, 40]. When cell density is decreased, ZONAB is activated and transferred to the nucleus as a transcription factor [41]. Expression levels of ZONAB mRNA appeared higher in hepatocellular carcinoma (HCC) than that in corresponding nontumor tissues [42]. ZONAB expression was increased in gastric cancer which acts as a significant part in its pathogenesis and development [43]. Our result indicated that there was overexpression of ZONAB in BC, and high ZONAB expression was positively correlated with muscle invasion of BC, which is consistent with the results of Liu RT and his colleagues' study in colorectal cancer [44]. The tumor-promoting role of ZONAB might be caused by its activation and binding to p21 mRNA to enhance cell survival [45]. Since ZO-1 and ZONAB are critical modulators determining the switching time for epithelial cells along proximal tubules changing from a proliferative (nuclear/cytoplasmic ZONAB) state to a differentiated (ZO-1-bound ZONAB) one [46], we monitored the high expression rate of ZO-1 and ZONAB in BC and observed a negative correlation between their expression, which means that ZO-1 and ZONAB interaction might be involved in BC development.

## 5. Conclusion

In summary, the expression of tight junction proteins was significantly altered in BC. The expression of ZO-1 and ZONAB might be involved in muscle invasion of BC lesions and ZO-1, and ZONAB interaction might be involved in BC development. Further study is required for proving the invasion-promoting character of ZO-1, for ZONAB interaction both in vitro and in vivo, and for revealing the potential signaling pathway by which these tight junction proteins regulate the invasion and metastasis of BC.

## Figures and Tables

**Table 1:** Correlation between claudin-4, ZO-1, and ZONAB expressions and clinicopathological factors of bladder cancer detected by immunohistochemical test.

Index	*n*	High expression					
		Claudin-4 (%)	*P*	ZO-1 (%)	*P*	ZONAB (%)	*P*
Gender							
Male	42	18 (42.9)	0.880	25 (59.5)	0.808	16 (38.1)	0.918
Female	11	5 (45.5)		7 (63.6)		4 (36.4)	
Age (years)							
>65	36	15 (41.7)	0.718	22 (61.1)	0.877	11 (30.6)	0.121
≤65	17	8 (47.1)		10 (58.8)		9 (52.9)	
Muscle invasion							
+	23	11 (47.8)	0.578	10 (43.5)	0.028^a^	14 (60.9)	0.002^b^
−	30	12 (40.0)		22 (73.3)		6 (20.0)	
Lymph node metastasis							
+	9	2 (22.2)	0.166	4 (44.4)	0.292	2 (22.2)	0.301
−	44	21 (47.7)		28 (63.6)		18 (40.9)	
Distant metastasis							
+	3	1 (33.3)	0.412	2 (66.7)	0.823	1 (33.3)	0.874
−	50	22 (44.0)		30 (60.0)		19 (38.0)	

^a^Spearman's rank order correlation coefficient: -0.303; there is a negative correlation (*P* < 0.05). ^b^Spearman's rank order correlation coefficient: 0.418; there is a positive correlation (*P* < 0.05).

## Data Availability

All data generated or analyzed during this study are included in this article.
